# Quantifying Phosphorus and Water Demand to Attain Maximum Growth of *Solanum tuberosum* in a CO_2_-Enriched Environment

**DOI:** 10.3389/fpls.2019.01417

**Published:** 2019-11-05

**Authors:** Yan Yi, Daisuke Sugiura, Katsuya Yano

**Affiliations:** Graduate School of Bioagricultural Sciences, Nagoya University, Nagoya, Japan

**Keywords:** elevated CO2, critical phosphorus concentration, plant growth, leaf phosphorus, potato, water-use efficiency, starch

## Abstract

Growth promotion by ambient CO_2_ enrichment may be advantageous for crop growth but this may be influenced by soil nutrient availability. Therefore, we quantified potato (*Solanum tuberosum* L.) growth responses to phosphorus (P) supply under ambient (a[CO_2_]) and elevated (doubled) CO_2_ concentration (e[CO_2_]). A pot experiment was conducted in controlled-environment chambers with a[CO_2_] and e[CO_2_] combined with six P supply rates. We obtained response curves of biomass against P supply rates under a[CO_2_] and e[CO_2_] (R^2^ = 0.996 and R^2^ = 0.992, respectively). A strong interaction between [CO_2_] and P was found. Overall, e[CO_2_] enhanced maximum biomass accumulation (1.5-fold) and water-use efficiency (WUE) (1.5-fold), but not total water use. To reach these maxima, minimum P supply rate at both [CO_2_] conditions was similar. Foliar critical P concentration (i.e., minimum [P] to reach 90% of maximum growth) was also similar at nearly 110 mg P m^−2^. Doubling [CO_2_] did not increase P and water demand of potato plants, thus enabling the promotion of maximum growth without additional P or water supply, but *via* a significant increase in WUE (9.6 g biomass kg^−1^ water transpired), presumably owing to the interaction between CO_2_ and P.

## Introduction

Global atmospheric carbon dioxide concentration ([CO_2_]) has risen from 280 ppm in pre-industrial times to 400 ppm at present, and it is predicted to continue rising (Stocker et al., 2013). Despite serious concerns that increasing [CO_2_] will cause global warming, it might also have the potential to enhance crop production. This is because CO_2_ is the substrate for photosynthesis in plants, and the photosynthetic rate is not yet saturated under current [CO_2_], particularly in C3 plants. Previous studies showed that crop growth, especially in C3 plants, can be enhanced by the elevated concentration of atmospheric CO_2_ (e[CO_2_]) (Kimball, 1983; Figueiredo et al., 2015; Kimball, 2016; van der Kooi et al., 2016). However, it is frequently pointed out that such potential crop growth enhancement is likely to be limited by the downregulation of photosynthetic capacity, presumably due to the insufficient capacity of sink organs to use or store the increased carbohydrates (Ainsworth et al., 2004; Ruiz-Vera et al., 2017). Further, the accumulation of carbohydrates (e.g., starch) in source leaves due to the limited sink capacity (e.g., stimulation of phloem transport to sink organs) at e[CO_2_] could lead to a direct inhibition of photosynthesis (Stitt, 1991). Consistently, a large sink capacity has generally been proposed as a critical factor for maximizing plant production under e[CO_2_] (Marschner, 1995). In a study on five species, downregulation of photosynthesis was observed in four of the species studied, but not in potato (*Solanum tuberosum* L.) (Sage et al., 1989), suggesting that the large sink capacity of potato tubers may entail a high potential for potato yield enhancement under e[CO_2_].

Another critical factor that may limit plant production under e[CO_2_] is soil nutrient availability (Rogers et al., 1999; Lenka and Lal, 2012). Potato production is fertilizer intensive, requiring relatively large amounts of nutrients including phosphorous (P) (Singh, 1987; Alvarez‐Sánchez et al., 1999). Indeed, P deficiency has been reported to cause starch accumulation by affecting the photosynthetic electron transport chain (Carstensen et al., 2018). Although there are numerous studies on potato response to P fertilizer, only a few considered the possible interaction of [CO_2_] and P nutrition (Fleisher et al., 2012; Fleisher et al., 2013). Despite the numerous reports on the interactions between nutrients and [CO_2_] in various plants (de Graaff et al., 2006), the amount of each nutrient, including P, required for maximum plant growth under e[CO_2_] remains largely unknown. This is because most previous studies examined this issue at only two levels (high and low) of nutrient supply, neglecting maximum growth and its saturation. Furthermore, plant P status is a direct indicator for evaluating P nutrition and fertilizer management. One of such indices is critical P concentration ([P]), defined as the minimum [P] in the crop required to reach 90% of maximum growth (Chisholm et al., 1981; Conroy, 1992). Critical [P] reportedly became higher at e[CO_2_] in cotton and wheat (Rogers et al., 1993). However, it has not yet been examined if critical [P] is affected by [CO_2_] in potato plants. Although Fleisher et al. (2013) showed that P supply requirement for growth was unchanged by e[CO_2_] in potato plants, critical [P] was not investigated in their research. Hence, more rigorous and quantitative data covering the range from P-deficient to P-sufficient as growth rate becomes saturated, are needed to further assess e[CO_2_] effects and to develop appropriate P management in potato cropping.

Another important factor which may affect crop production at e[CO_2_] is water demand. Terrestrial plants acquire CO_2_ and lose water *via* stomata. Stomatal closure increases water-use efficiency (WUE) due to reduced water loss at the expense of CO_2_ acquisition, commonly resulting in poorer growth. Despite the disadvantage of e[CO_2_] regarding global warming, it may actually allow increased WUE without growth reduction, especially in C3 plants (Brouder and Volenec, 2008). An important question is how e[CO_2_] can affect water demand of crop plants, as approximately 70% of the fresh water consumed globally is used in agriculture (Clarke and King, 2004). Interestingly, it has been anticipated that e[CO_2_] will increase WUE by decreasing stomatal conductance and increasing assimilation rate (Polley, 2002; Ainsworth and Rogers, 2007). In addition, WUE was enhanced by P fertilizer under a[CO_2_] (Farahani et al., 2008; Sun et al., 2015). There are few reports on the effect of [CO_2_] on WUE under different P conditions. These include *Pinus radiata* (Conroy et al., 1988), grasses, legumes and forbs (Grünzweig and Körner, 2003), and pea (Jin et al., 2014), but not potato; hence, how P supply affects WUE under e[CO_2_] in potato plants remains unclear. To address the above questions, it is necessary to measure daily water consumption by the plant under different P supply and [CO_2_] conditions, and then estimate productive WUE as biomass increase per unit water used (i.e. accumulated daily water consumption) at the individual plant level.

In the present study, we examined the interaction between [CO_2_] and P supply on biomass production and water economy in potato plants. The aim was to quantify the growth response of the potato plant to P supply rate under two different [CO_2_] conditions to know how much P and water are required for maximum growth under each [CO_2_] condition. The following three questions were addressed: 1) to what extent can maximum biomass accumulation be enhanced by e[CO_2_] in potato plants?, 2) how much P is required to achieve maximum biomass accumulation and, will e[CO_2_] increase the plant P requirement?, and finally 3) how does e[CO_2_] affect water consumption by the plant to reach maximum biomass accumulation under varying P supply?

## Materials and Methods

### Experimental Design and Growing Conditions

A pot experiment was undertaken in controlled-environment chambers (LPH-410 SPC, Nippon Medical & Chemical Instruments Co., Ltd., Japan). The environmental conditions inside the chambers were set as follows: light intensity, 400 µmol m^−2^ s^−1^; temperature, 25/17°C day/night, respectively; and photoperiod, 14/10 h day/night, respectively. Relative humidity was not controlled, but it was approximately 36%/48% day/night, respectively. The CO_2_ concentrations were controlled at approximately 400 ppm for a[CO_2_] and 800 ppm for e[CO_2_]. The plants and CO_2_ concentrations were switched weekly between the two chambers to minimize any potential chamber effects. A CO_2_ recorder (TR-76Ui, T&D Inc., Japan) was placed inside each chamber to monitor practical conditions in the chambers every 10 min (**Figure S1**).

Naturally sprouted potato tubers (cv. "Irish Cobbler") were transplanted into 1-L pots (diameter, 11.3 cm; depth, 14 cm; one plant per pot) filled with 580 g of dry andosol. Before transplanting, nitrogen (0.4 g N kg^−1^ dry soil) and potassium (0.4 g K_2_O kg^−1^ dry soil) were mixed with the soil in the form of ammonium sulfate (21.0% N) and potassium chloride (60.0% K_2_O), respectively. At 24 and 40 days after transplanting, respectively, 0.77 g KNO_3_ dissolved in tap water was added to each pot to provide enough N and K for plant growth. Calcium superphosphate (17.5% P_2_O_5_) was uniformly mixed with the soil to control P levels at 0, 0.4, 0.8, 1.2, 2.4, or 3.6 g P kg^−1^ of dry soil (hereafter, these treatments are designated as P0, P0.4, P0.8, P1.2, P2.4, and P3.6, respectively). Soil water condition was kept at 60% (w/w) by adding tap water to each pot to compensate for water-loss due to transpiration until 40 days after transplanting, and then kept at 80% (w/w) until harvest to avoid drought stress. The experiment was organized following a factorial design (two CO_2_ concentrations × six P supply rates) with six biological replications.

### Measurement of Water Use

A transparent plastic film was used to cover each pot to prevent water loss by soil evaporation. Because the pots used had no holes in the bottom, leaching was not considered. We weighed the pots every 2–4 days until 42 days after transplanting and then every day until harvest before watering. A decrease in pot weight was regarded as water consumption by transpiration, and the same amount of water lost by transpiration was provided to each pot. The pot weight and the amount of water given to each pot were recorded throughout the growth period. Water use during the growth period was calculated from cumulative transpiration. WUE was calculated as total plant biomass/water use according to Jones (2004).

### Harvest and Sampling

All plants were harvested 54 days after transplanting. Before harvest, three young fully expanded leaves were sampled from each plant to ensure sample size was sufficient for starch and P quantification (see below). Sampled leaves were immediately frozen in liquid N and then dried for starch and P analysis. At harvest, the remaining leaves, stems, roots, and tubers were separated and dried in an oven at 80°C to constant mass for dry weight determination. All samples were then ground to powder for P quantification. Soil samples were collected after harvesting and then dried at 80°C to constant mass for available P and pH analysis. Leaf area and root morphological parameters (root length and root surface area) were analyzed in a flatbed scanner (EPSON EXPRESSION 10000XL, Seiko Epson Co., Japan) using software WinRHIZO Pro LA2400 (Regent Instruments Inc., Canada) before drying.

### Stomatal Conductance and Stomatal Density

On harvest day, stomatal conductance of the youngest fully expanded leaf was measured on the adaxial surface at 8:00-12:00 in the morning with a leaf porometer (SC-1, Decagon Devices Inc., USA). Immediately after measurement of stomatal conductance, the same leaves were coated with nail polish; next, imprints were taken from each leaf and mounted on a glass microscope slide to count the number of stomata under the microscope (SZ61, OLYMPUS Co., Tokyo, Japan). Five observations of each imprint were randomly selected to count the number of stomata; thus, data presented are means of five individual measurements per leaf.

### Starch Quantification

Starch content was determined after Ono et al. (1996). Samples (3–5 mg each) of micro-ground dried tissue from young leaves were placed in 2-ml microtubes containing 0.75 ml 80% ethanol and heated at 78.5°C for 10 min on a heating block. The supernatants were transferred after centrifugation (12,000 g, room temperature, 10 min) and 0.5 ml 80% ethanol was added into each tube to heat at 78.5°C for 10 min once again. After centrifugation at 18,000 g, at room temperature for 10 min, the supernatants were transferred and residues containing starch were dissolved in 400 µl Milli-Q water, heated at 98°C for 1 h, and then cooled to room temperature. After adding 400 µl amyloglucosidase (70 units G-amyloglucosidase/ml 50 mM Na-acetate buffer at pH 4.5), samples were incubated at 55°C for 1 h. After digestion of starch to glucose, samples were centrifuged at 18,000 g, at room temperature for 10 min, and the supernatants were then assayed for glucose using a Glucose CII Test Kit (Wako Chemicals, Tokyo, Japan). The assay reagents were mixed into the samples and the reaction was incubated for 10 min at room temperature before measuring their absorption at 505 nm (A_505_) in a microplate reader (Sunrise Rainbow Thermo, Tecan Japan, Co., Ltd., Japan).

### Phosphorus Quantification in Plant Material

Plant tissue P concentrations were determined according to Watanabe and Olsen (1965). We determined [P] in the young leaves that were also used for starch quantification, and in the remaining leaves, stems, tubers, and roots. Dried samples (40−60 mg) were weighed into crucibles and ashed at 495°C for 2 h. After cooling to room temperature, 1 ml 4 M HCl was added to the crucibles and then transferred to 25-ml volumetric flasks. The distilled water used to wash the crucibles for three times was also transferred to the same volumetric flasks, and identical volumes across flasks were obtained by adding distilled water. An appropriate amount of sample solution was transferred to a new 25-ml volumetric flask, and 4 ml color-substrate solution (2.5 M H_2_SO_4_:4% (NH_4_)_6_Mo_7_O_24_·4H_2_O:10 M C_6_H_8_O_6_:0.44 M= 10:3:6:1) was added first, followed by distilled water to constant volume. The solutions were mixed and incubated at room temperature for 15 min; next, absorbance at 710 nm (A_710_) was measured using a UV spectrophotometer (UV-1800, Shimadzu Inc., Japan).

### Soil Available P After Harvest

Dried, 1-g soil samples were weighed into 200-ml bottles. Next, 200 ml 1 mM H_2_SO_4_ (pH 3.0) was added to each bottle. Extracts were filtered through an ashless paper after oscillation for 30 min. The filtrates were used to measure available P according to the method described above for P quantification in plant organs.

### Soil pH After Harvesting

Dried, 10-g soil samples were weighed into 50-ml tubes; next, 25 ml distilled water was added to each tube. After vigorous stirring, tubes were allowed to stand for 30 min. A pH meter (LAQUAact D-73, HORIBA Inc., Japan) was used to measure the pH of the soil solution.

### Statistical Analysis

The experiment was organized following a factorial design with two CO_2_ concentrations and six P supply rates with six replications (except for the e[CO_2_] P3.6 treatment, as one plant in this treatment died after transplanting), data are expressed as mean ± standard error (S.E.) for six (or five) biological replicates. Data were analyzed in SPSS 16.0 (SPSS Inc., Chicago, IL, USA) using two-way analysis of variance (ANOVA) at the 0.05 probability level. Simple regressions were analyzed in Origin 9.0 (https://www.originlab.com). Coefficients of the exponential equations (y = a – b × *c* *^x^*) from regression relationships between foliar [P] and total biomass were used to calculate critical [P] (critical [P] = Log (a/10b, c)). Foliar [P] was defined as the [P] of all leaves, including young leaves and remaining leaves. P-acquisition efficiency was calculated after Boucho et al. (2019).

$$\begin{array}{l} \text{P}-\text{acquisition efficiency}\\ \frac{=(\text{P content in P}\_\text{treated plants}-\text{P content in P}0\text{ plants})}{\text{P supply rate}} \end{array}$$

## Results

### Plant Growth and Biomass Partitioning

**Figure 1:**
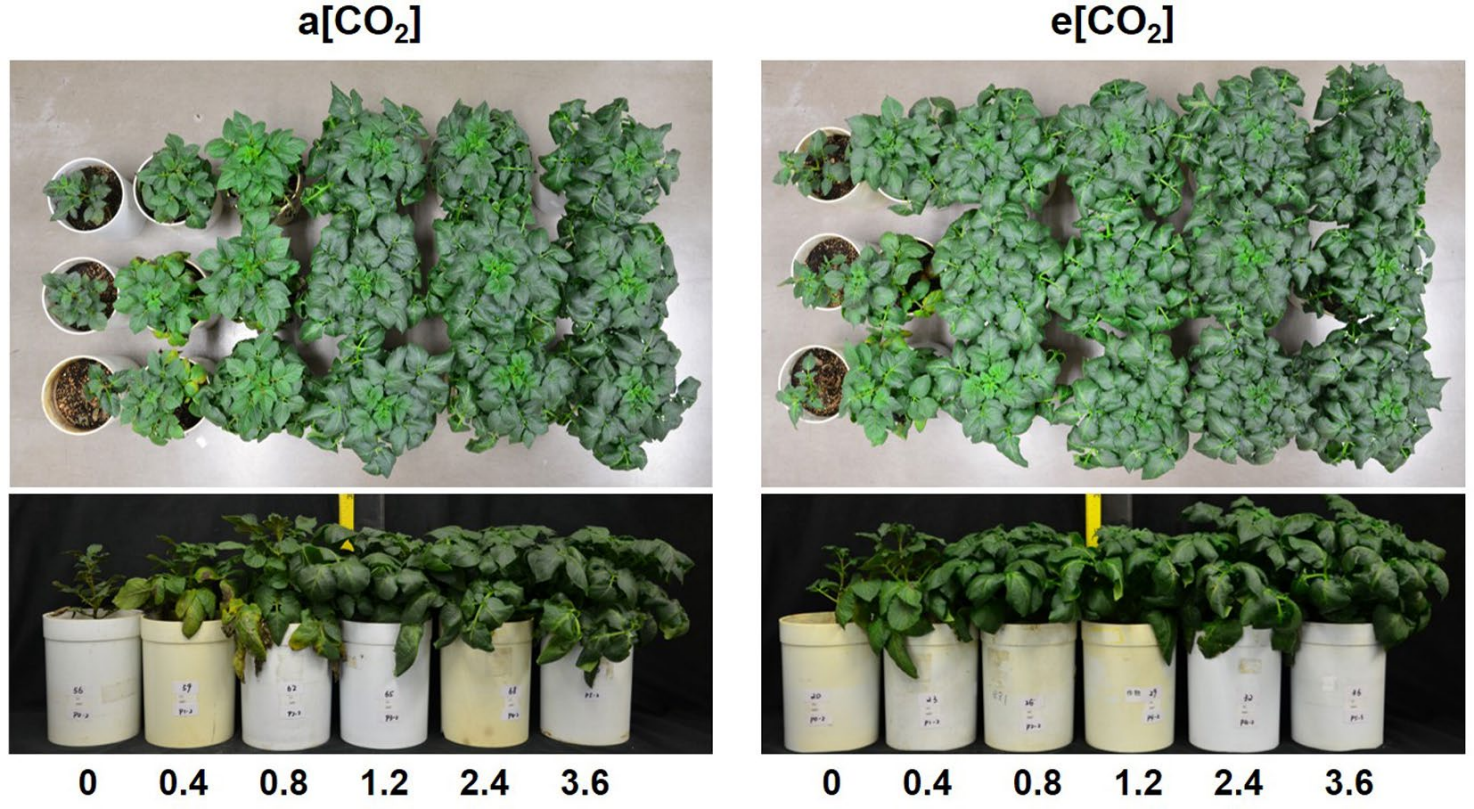
Appearance of potato plants at harvest (54 days after transplanting).

**Figure 2:**
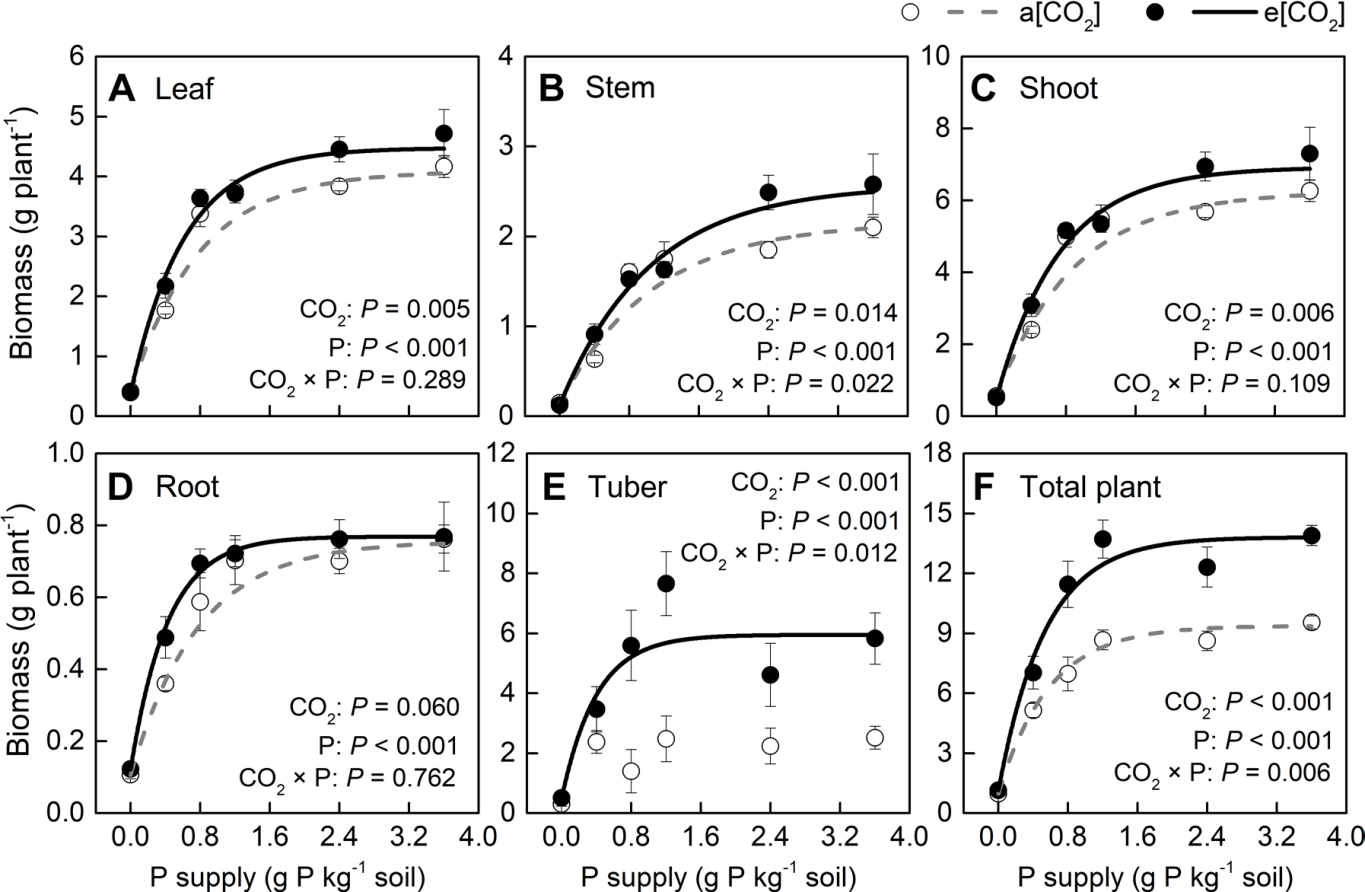
Biomass of several organs of potato plants grown under a[CO_2_] (439 ± 9 ppm) and e[CO_2_] (825 ± 17 ppm) at different P supply rates (0, 0.4, 0.8, 1.2, 2.4, and 3.6 g P kg^−1^ soil). Data in each plot are means ± S.E. (n = 6 or 5 biological replicates for each treatment). Statistical comparisons (two-way ANOVA) between CO_2_ concentrations and P supply rates as well as their interaction (CO_2_ × P) are presented. **(A)** Leaf biomass; **(B)** stem biomass; **(C)** shoot biomass; **(D)** root biomass; **(E)** tuber biomass; and **(F)** total plant biomass. Regressions are as follows: **(A)** a[CO_2_]: y = 4.088 – 3.694 × 0.279*^x^*, R^2^ = 0.982; e[CO_2_]: y = 4.475 – 4.083 × 0.197*^x^*, R^2^ = 0.992. **(B)** a[CO_2_]: y = 2.167 – 2.026 × 0.396*^x^*, R^2^ = 0.939; e[CO_2_]: y = 2.570 – 2.444 × 0.382*^x^*, R^2^ = 0.988. **(C)** a[CO_2_]: y = 6.252 – 5.719 × 0.328*^x^*, R^2^ = 0.971; e[CO_2_]: y = 6.924 – 6.406 × 0.253*^x^*, R^2^ = 0.990. **(D)** a[CO_2_]: y = 0.756 – 0.650 × 0.276*^x^*, R^2^ = 0.991; e[CO_2_]: y = 0.769 – 0.647 × 0.095*^x^*, R^2^ = 0.998. **(E)** a[CO_2_]: no fitting curve; e[CO_2_]: y = 5.947 – 5.446 × 0.082*^x^*, R^2^ = 0.924. **(F)** a[CO_2_]: y = 9.378 – 8.403 × 0.179^x^, R^2^ = 0.996; e[CO_2_]: y = 13.808 – 12.670 × 0.156*^x^*, R^2^ = 0.992.

Based on plant appearance, soil P availability obviously limited growth below P1.2 under a[CO_2_], while at P0.8 growth seemed to be similar to that at P1.2 under e[CO_2_] (**Figure 1**). Plants were harvested at 54 days after transplanting. In terms of dry weight, e[CO_2_] significantly increased leaf, stem, and tuber, but not root biomass (**Figure 2**). As for P rates, total biomass increased with increasing P supply rates and reached a maximum at 1.2 g P kg^−1^ soil under both [CO_2_] conditions tested (**Figure 2F**). There was a significant effect from the interaction between [CO_2_] and P supply rate on total plant biomass (*P* = 0.006), tuber biomass (*P* = 0.012), and stem biomass (*P* = 0.022). Maximum plant total biomass was enhanced by 47% under e[CO_2_] compared to a[CO_2_]; however, e[CO_2_] did not alter P supply requirement for total plant maximum biomass (**Figure 2F**).

**Figure 3:**
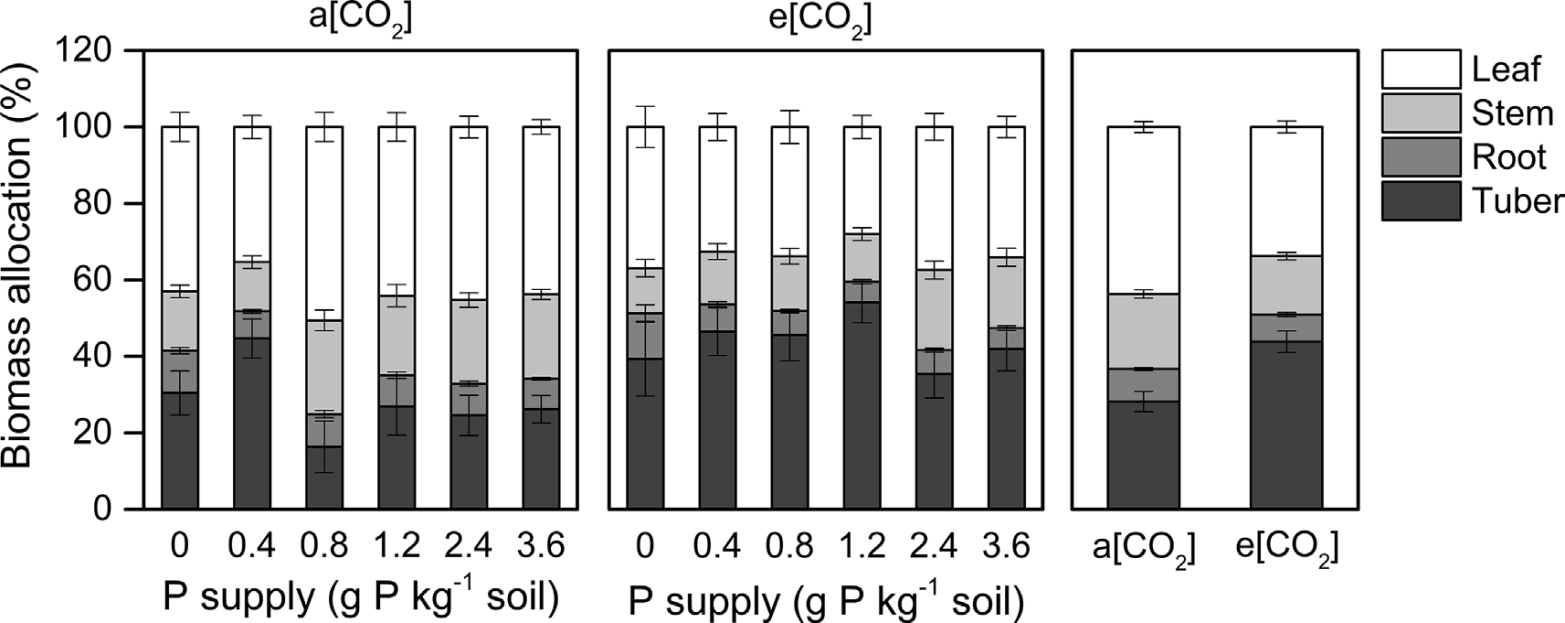
Biomass partitioning in organs of potato plants grown under a[CO_2_] (439 ± 9 ppm) and e[CO_2_] (825 ± 17 ppm) at different P supply rates (0, 0.4, 0.8, 1.2, 2.4, and 3.6 g P kg^−1^ soil). Data in each plot are means ± S.E. (n = 6 or 5 biological replicates for each treatment).

Dry matter assimilation and partitioning are important processes determining crop productivity. Our study suggested that e[CO_2_] enhanced tuber growth (**Figure 3**). Thus, at harvest, the dry matter proportion in the leaf (44 ± 1.5%) was highest under a[CO_2_]. However, a greater proportion of biomass was allocated to tubers (44 ± 2.8%) under e[CO_2_] at the expense of dry matter accumulation in stems, roots, and leaves. As for P effects, the largest P rates reduced the proportion of dry matter allocated to tubers at P0.8 and P2.4 under a[CO_2_] and e[CO_2_], respectively, likely due to shoot overgrowth induced by high P supply.

**Figure 4:**
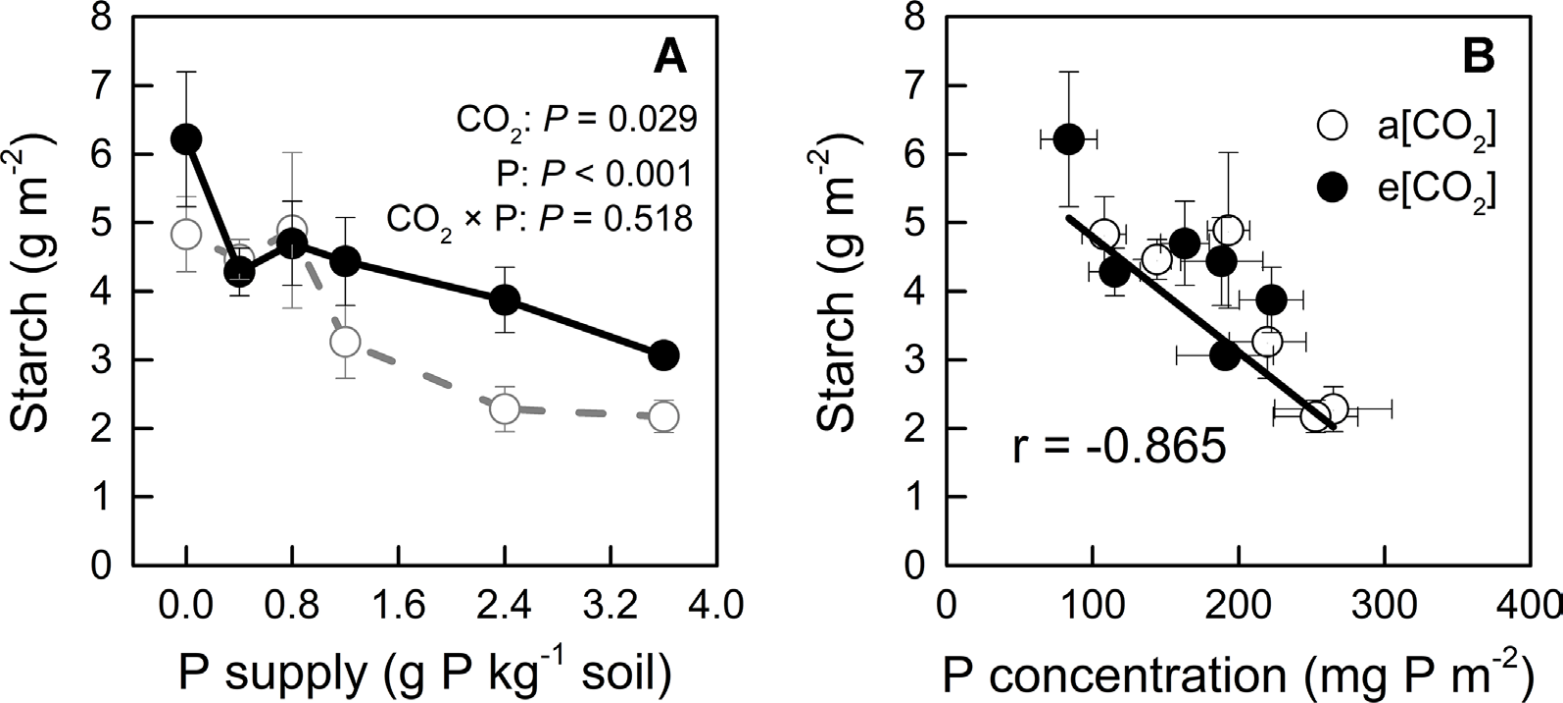
**(A)** Starch concentration in young leaves under a[CO_2_] (439 ± 9 ppm) and e[CO_2_] (825 ± 17 ppm) at different P supply rates (0, 0.4, 0.8, 1.2, 2.4, and 3.6 g P kg^−1^ soil). Statistical comparisons (two-way ANOVA) between CO_2_ concentrations and P supply rates as well as their interaction (CO_2_ × P) are presented. **(B)** Relationship between starch concentration and P concentration in the young leaves under a[CO_2_] and e[CO_2_]. Data in each plot are means ± S.E. (n = 6 or 5 biological replicates for each treatment).

On the other hand, e[CO_2_] and P deficiency increased starch accumulation in young leaves (**Figure 4A**), which could further reduce CO_2_ assimilation (Nakano et al., 2000); indeed, P deficiency has been reported to cause starch accumulation by affecting photosynthetic electron transport chain (Carstensen et al., 2018). Our study showed that starch concentration correlated negatively with [P] in young leaves, regardless of CO_2_ conditions (**Figure 4B**); this finding suggested that increased starch accumulation under e[CO_2_], especially at higher P supply rates, could be related to reduced [P].

### Water Use and Water-Use Efficiency

**Figure 5:**
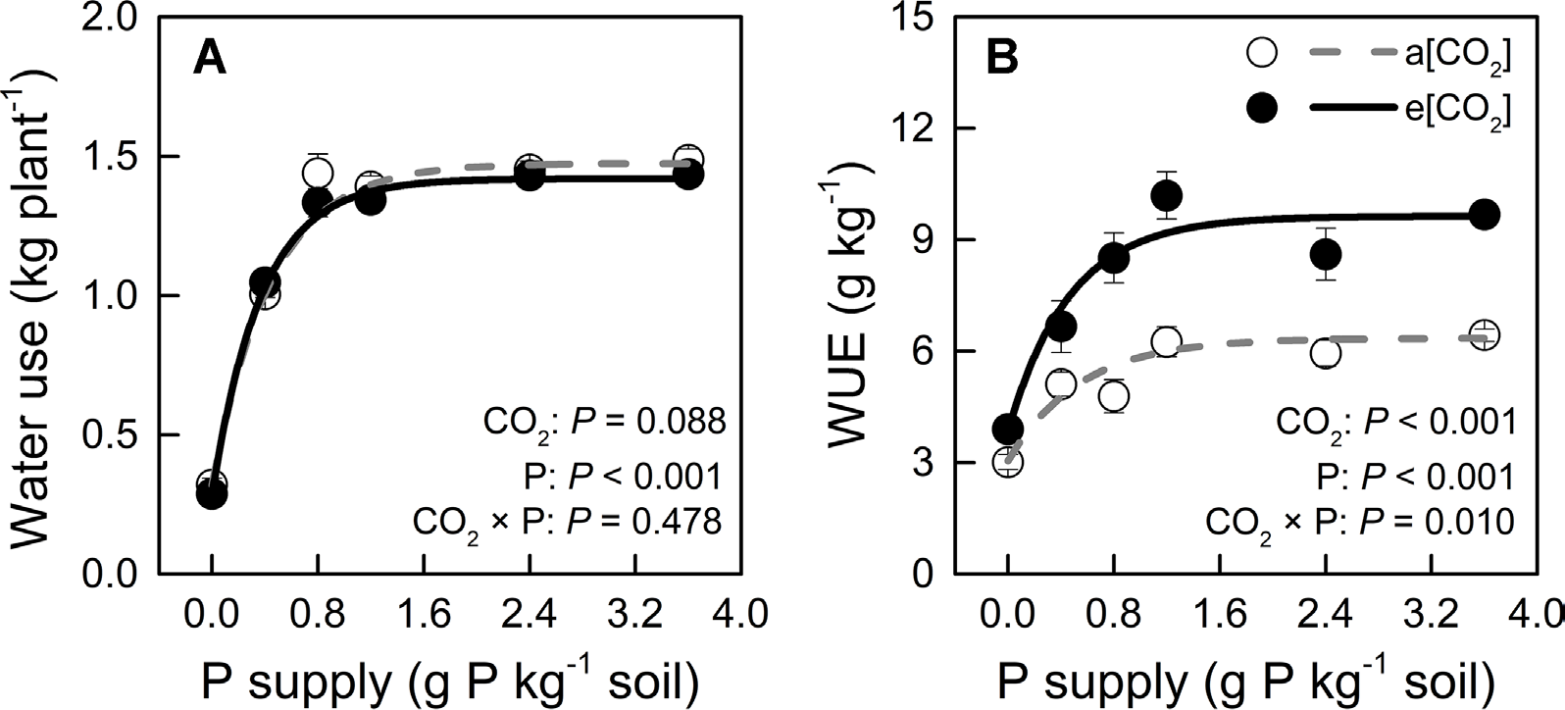
**(A)** Water use (during the 54 days after transplanting) and **(B)** water-use efficiency of potato plants grown under a[CO_2_] (439 ± 9 ppm) and e[CO_2_] (825 ± 17 ppm) at different P supply rates (0, 0.4, 0.8, 1.2, 2.4, and 3.6 g P kg^−1^ soil). Data in each plot are means ± S.E. (n = 6 or 5 biological replicates for each treatment). Statistical comparisons (two-way ANOVA) between CO_2_ concentrations and P supply rates as well as their interaction (CO_2_ × P) are presented. Regressions are as follows: **(A)** a[CO_2_]: y = 1.474 – 1.155 × 0.104*^x^*, R^2^ = 0.993; e[CO_2_]: y = 1.419 – 1.130 × 0.070^x^, R^2^ = 0.997. **(B)** a[CO_2_]: y = 6.349 – 3.303 × 0.154^x^, R^2^ = 0.944; e[CO_2_]: y = 9.632 – 5.758 × 0.119^x^, R^2^ = 0.965.

We monitored time-course changes in cumulative transpiration as water use in potato plants (**Figure S2**). Water use was unaffected by e[CO_2_] at each P level (**Figure 5A**). The significant increase in WUE under e[CO_2_] might be attributed to biomass increase (**Figures 2F** and **5**). Water use increased with increasing P supply rate and peaked at P0.8 under both CO_2_ conditions tested. Similarly, WUE was enhanced by increasing P supply rate and reached its maximum at P1.2 under both CO_2_ conditions tested. The two-way ANOVA showed that there was a significant interaction (*P* = 0.010) between CO_2_ and P supply rates on WUE (g kg^−1^), indicating that WUE increased with e[CO_2_] in a P supply-dependent manner.

**Figure 6:**
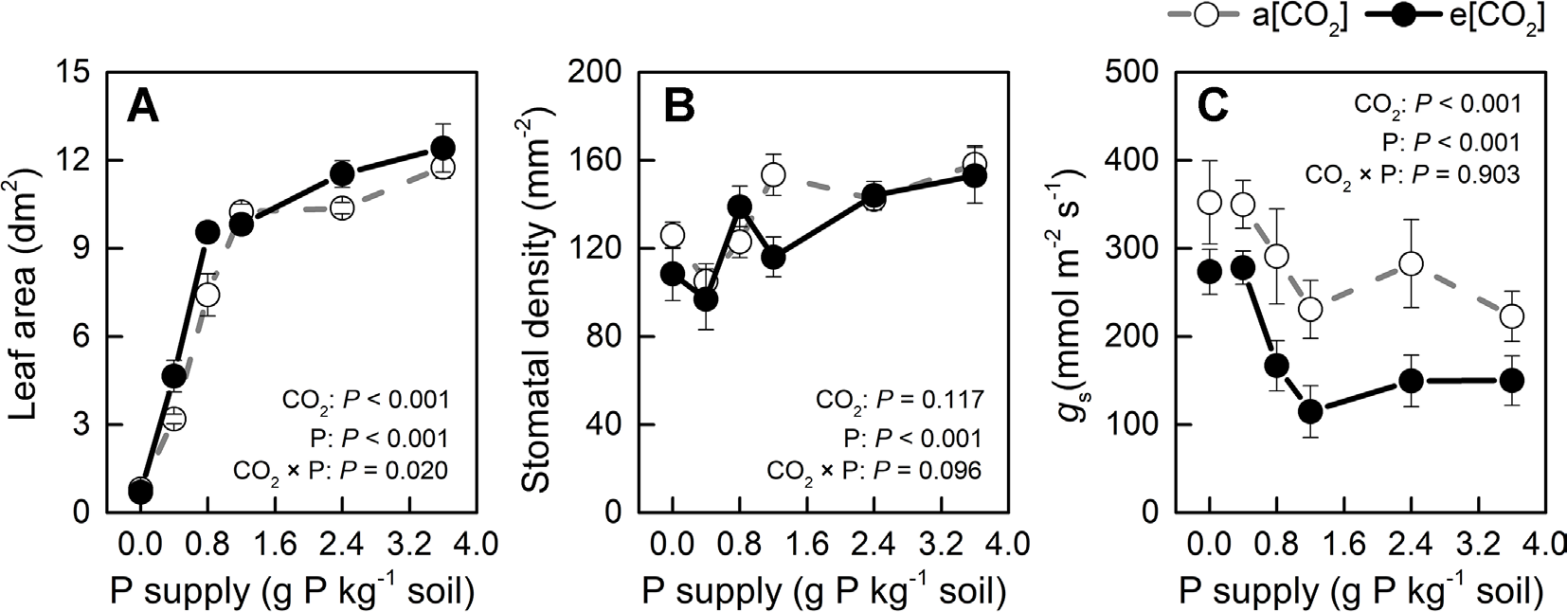
**(A)** Leaf area, **(B)** stomatal density, and **(C)** stomatal conductance of the youngest fully expanded leaf under a[CO_2_] (439 ± 9 ppm) and e[CO_2_] (825 ± 17 ppm) at different P supply rates (0, 0.4, 0.8, 1.2, 2.4, and 3.6 g P kg^−1^ soil). Data in each plot are means ± S.E. (n = 6 or 5 biological replicates for each treatment). Statistical comparisons (two-way ANOVA) between CO_2_ concentrations and P supply rates as well as their interaction (CO_2_ × P) are presented.

Leaf area was slightly larger in e[CO_2_] than in a[CO_2_] at 0.4, 0.8, and 2.4 P supply levels and similar among the remaining treatments (**Figure 6A**) while P levels had a major effect on this parameter. Therefore, unchanged water use by e[CO_2_] could be related to stomatal density or stomatal conductance. As for stomatal density, there was no significant difference between a[CO_2_] and e[CO_2_], but it increased with increasing P supply (**Figure 6B**). As **Figure 6C** shows, e[CO_2_] largely decreased stomatal conductance compared to a[CO_2_]; further, stomatal conductance decreased with increasing P supply and reached a minimum at P1.2 under both [CO_2_] conditions. Therefore, increased WUE under e[CO_2_] was likely related to reduced stomatal conductance, which was also affected by P supply level.

### Critical [P]

**Figure 7:**
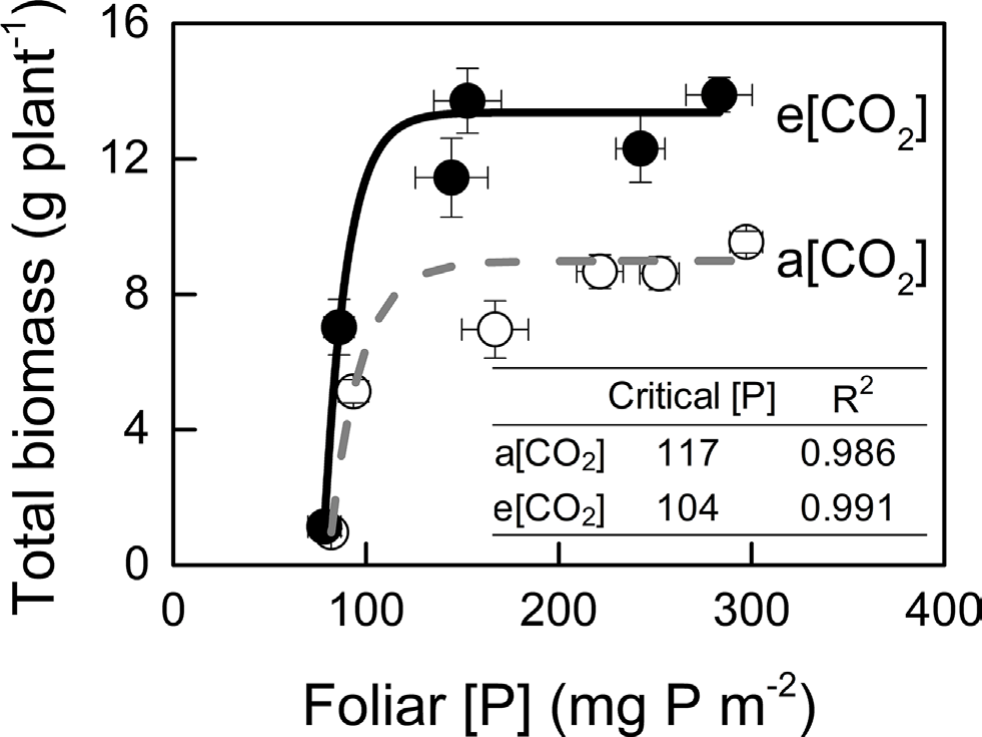
Relationships between foliar P concentration and total biomass. Critical P concentration is defined as the minimum concentration of P required by the crop to reach 90% of maximum growth. Critical P concentrations and R^2^ values for regressions are presented. Data in each plot are means ± S.E. (n = 6 or 5 biological replicates for each treatment). Regressions are as follows: a[CO_2_]: y = 8.985 – 1392.014 × 0.939*^x^*, R^2^ = 0.986; e[CO_2_]: y = 13.375 – 9835.24 × 0.918*^x^*, R^2^ = 0.991.

We assessed critical [P] in potato plants because P demand by plant under e[CO_2_] are likely to increase due to the stimulation of photosynthesis (Jin et al., 2015). In the present study, critical [P] was similar under a[CO_2_] (117 mg P m^−2^) and e[CO_2_] (104 mg P m^−2^) (**Figure 7**).

**Figure 8:**
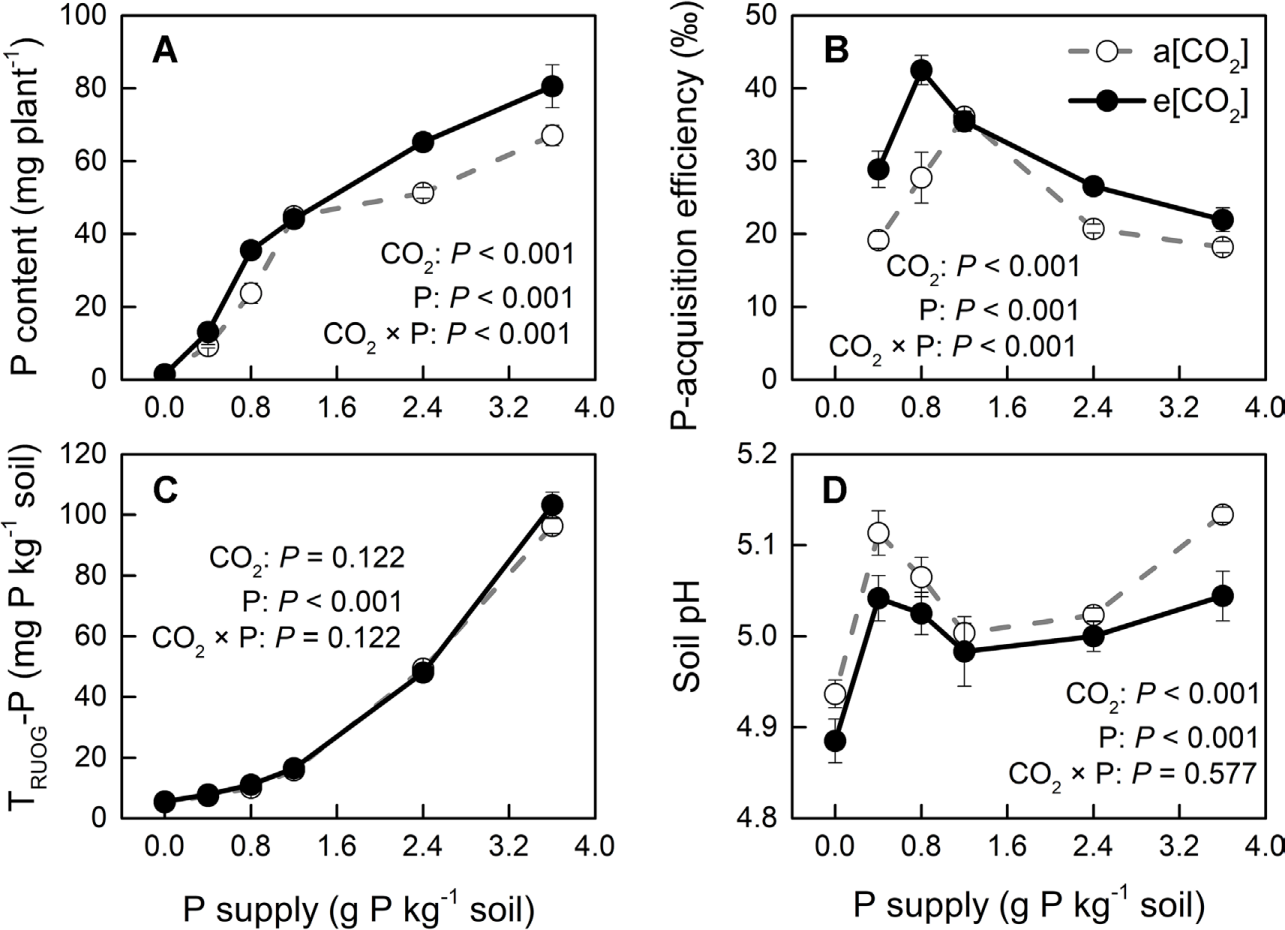
**(A)** Total plant P content, **(B)** P-acquisition efficiency, **(C)** soil T_RUOG_-P, and **(D)** soil pH after harvesting under a[CO_2_] (439 ± 9 ppm) and e[CO_2_] (825 ± 17 ppm) at different P supply rates (0, 0.4, 0.8, 1.2, 2.4, and 3.6 g P kg^−1^ soil). Data in each plot are means ± S.E. (n = 6 or 5 biological replicates for each treatment). Statistical comparisons (two-way ANOVA) between CO_2_ concentrations and P supply rates as well as their interaction (CO_2_ × P) are presented. P-acquisition efficiency was calculated as: (P content in P treated plant − P content in P0 plant)/P input rate.

**Figure 9:**
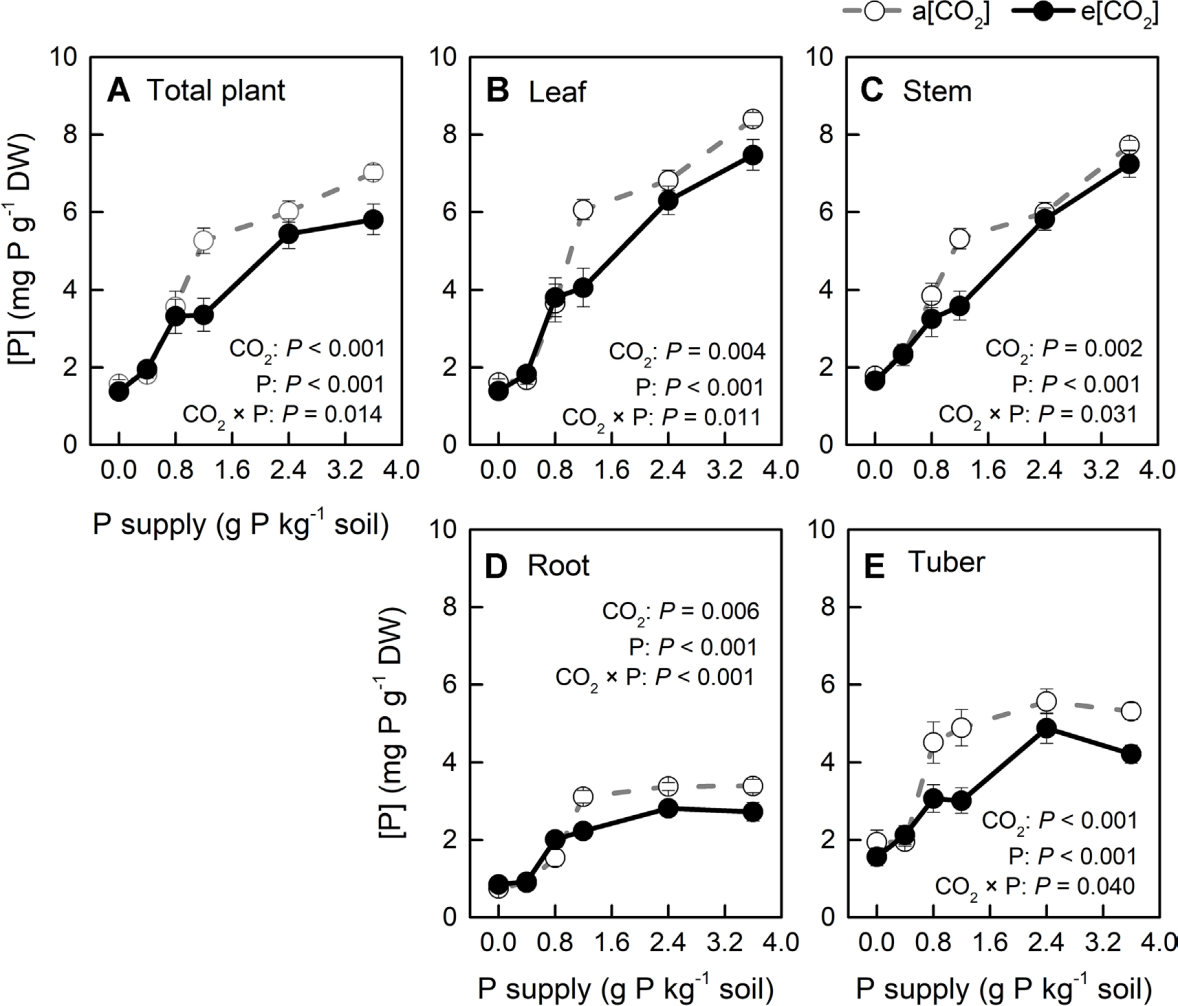
P concentration ([P]) in organs of potato plants grown under a[CO_2_] (439 ± 9 ppm) and e[CO_2_] (825 ± 17 ppm) at different P supply rates (0, 0.4, 0.8, 1.2, 2.4, and 3.6 g P kg^−1^ soil). Data in each plot are means ± S.E. (n = 6 or 5 biological replicates for each treatment). Statistical comparisons (two-way ANOVA) between CO_2_ concentrations and P supply rates as well as their interaction (CO_2_ × P) are presented. **(A)** Total plant P concentration; **(B)** leaf P concentration; **(C)** stem P concentration; **(D)** root P concentration; **(E)** tuber P concentration.

Phosphorus-acquisition efficiency and P-utilization efficiency (1/[P]) in potato plants were enhanced by e[CO_2_] (**Figures 8B** and **9A**). Total plant P content was higher under e[CO_2_] than under a[CO_2_] (**Figure 8A**). e[CO_2_]-increased P content resulted from increased tuber P content, as P content was not affected by [CO_2_] in leaves, stems or roots (**Figure S3**). At harvest, root parameters were measured to evaluate root function for P uptake in potato plants. The results showed that root length was decreased, whereas root surface area was not changed by e[CO_2_] (**Figure S4**). Neither of these parameters could explain the increase in P-acquisition efficiency under e[CO_2_]. Due to the increased amount of P under e[CO_2_], soil available P (T_RUOG_-P) may be lower under e[CO_2_]. However, this result suggested that there was no significant difference in soil T_RUOG_-P between the two CO_2_ conditions (**Figure 8C**). In addition, soil pH under e[CO_2_] was lower than that under a[CO_2_] (**Figure 8D**). Thus, soil pH might contribute to sustain soil P availability, thereby enhancing P-acquisition efficiency under e[CO_2_].

[P] in all plant organs varied with CO_2_ conditions and P supply rates tested; further, two-way ANOVA results confirmed a strong interaction between these two factors (**Figure 9**). Additionally, [P] decreased in all plant organs and at the whole plant level under e[CO_2_], compared with a[CO_2_], but it was less affected at P0, P0.4, and P0.8. Clearly, [P] became higher with increasing P supply. Interestingly, [P] in the leaves (including young leaves and remaining leaves) and stems increased linearly with P supply (**Figures 9B, C**), whereas it increased nonlinearly in roots and tubers (**Figures 9D, E**). This could be explained by P distribution in the plant. Because P plays important roles in photosynthesis (Zhang et al., 2014; Carstensen et al., 2018), it is generally transported to the storage pools in vacuoles and other organelles, such as chloroplasts, where the photosynthetic machinery is located.

## Discussion

Tissue [P] in potato plants generally ranges between 1 to 4 mg P g^−1^ dry mass (Jenkins and Mahmood, 2003; White et al., 2018). In the present study, [P] varied among plant organs, varying from 0.75 to 8.40 mg P g^−1^ dry mass, depending on growth conditions (**Figure 9**). This wide range indicated that P supply levels fully covered P-deficient and P-sufficient conditions. P deficiency was reported to increase starch in leaves by affecting photosynthetic electron transport chain (Carstensen et al., 2018). Consistently, we detected a negative correlation of starch concentration with [P] in leaves across each [CO_2_] (**Figure 4B**), suggesting that starch accumulation responded more strongly to foliar [P] than to [CO_2_]. A similar consistency with previous researches (Ainsworth et al., 2002; Katny et al., 2005; Jauregui et al., 2018) was found for leaf starch, which increased under e[CO_2_] (**Figure 4A**), and may be explained by the decrease in [P] observed under e[CO_2_] (**Figure 4B**).

### 1.5-Fold Increase in Maximum Total Biomass by e[CO_2_] Followed a Phosphorus Supply Dependent Pattern

In contrast to Fleisher et al. (2012, 2013), who did not find a significant interaction between [CO_2_] and P supply in potato, our study clearly demonstrated significant [CO_2_]—P-supply interaction effects on organ biomass (**Figure 2**) and WUE (**Figure 5B**), but not on water use (**Figure 5A**). The difference between these contradictory results may be related to the growing conditions and potato cultivar used in these studies. The strong interaction in our results clearly indicated that e[CO_2_] effects were greatly dependent on P supply. Thus, maximum biomass enhancement by CO_2_ enrichment was evident only when P demand was fully supplied, an effect that involved enhanced WUE, but not water use. Moreover, both biomass and WUE clearly described a saturation-kinetics pattern when plotted against P supply, hence enabling an accurate estimation of minimum P supply rate to reach maximum performance. To our knowledge, this is the first report of a saturation response-curve against P supply rate under different CO_2_ conditions in potato plants. Therefore, maximum plant total biomass under e[CO_2_] was achieved even without additional P or water supply, despite the 1.5-fold biomass increase relative to biomass accumulation under a[CO_2_].

Because e[CO_2_] can also affect plant respiration (Gonzalez-Meler et al., 2004), carbon-use efficiency (CUE), which is defined as the ratio of net primary production to gross primary production, is important for understanding CO_2_-fertilization effects (Long et al., 2006). Gong et al. (2017) reported the reduced inhibition of leaf respiration by light and the diminished leaf mass ratio as the two main process contributing to the reduction of CUE under e[CO_2_]. Consistent with previous studies (Norby et al., 2004; Gong et al., 2017), the potato plants examined in this investigation developed a lower leaf (source) mass ratio and a higher tuber (sink) mass ratio (**Figure 3**). Although leaf respiration was not measured in our study, leaf starch concentration was higher under e[CO_2_] than under a[CO_2_] (**Figure 4A**). Amthor (2000) reported that accumulation of nonstructural carbohydrates in source leaves may stimulate respiratory activity through increased phloem loading and translocation. Therefore, CUE in potato plants could be decreased at e[CO_2_] through the decrease in leaf mass ratio and increase in leaf respiration, as detected in the present study.

### P Supply Requirement for Maximum Total Biomass Was Unchanged by e[CO_2_]

Generally, plants growing under e[CO_2_] are expected to require a larger P supply to take full advantage of the CO_2_ enrichment (Pang et al., 2018). Consistently, Rogers et al. (1993) found that, on a foliar mass basis, critical [P] increased under e[CO_2_] in cotton and wheat. By contrast, our data showed a similar critical [P] (approximately 110 mg P m^−2^) under the two [CO_2_] conditions tested (**Figure 7**). Although total P uptake was higher under e[CO_2_] compared to that under a[CO_2_] (**Figure 8A**), P supply requirement for maximum growth was unchanged by e[CO_2_] (**Figure 2F**) because of higher P-acquisition efficiency (**Figure 8B**) or P-use efficiency (1/[P]) (**Figure 9A**). Thus, the maximum biomass under both [CO_2_] was obtained at the same P supply rate (1.2 g P kg^−1^ soil) (**Figure 2F**). Notably, plant P content at P1.2 (which allowed maximum plant growth) was similar regardless of [CO_2_] (**Figure 8A**). Nevertheless e[CO_2_] allowed 1.5-fold higher plant total biomass than a[CO_2_], thus resulting in higher P-utilization efficiency (1/[P]) (**Figure 9A**).

Root growth and morphology greatly affect the amount of P extracted from the soil because of P bioavailability constraints. Silberbush and Barber (1983) demonstrated that in soils with low P availability, root length was an excellent predictor of P content in some plant species. However, in the present study, e[CO_2_] increased P-acquisition efficiency in potato plants not by increasing root length or root surface area (**Figure S4**). Therefore, we hypothesized that the increase in P-acquisition efficiency observed in the potato plants used here was likely due to a lowering of soil pH under e[CO_2_] through a change in the rhizosphere. In fact, rhizosphere acidification might increase the concentration of phosphate in the rhizosphere by increasing the desorption of phosphate from the soil solid phase (Hedley et al., 1982). Whether rhizosphere acidification might result from enhanced root or microbial activity remains unknown.

### e[CO_2_]-Induced Increase in Water-Use Efficiency Was P Dependent

Our results indicate that under e[CO_2_], WUE increased relative to a[CO_2_] in a P-dependent manner (**Figure 5B**). As drought stress is a serious limitation for plant growth in many cropping regions of the world, it is interesting that e[CO_2_] may be more than palliative to allow increased WUE without growth reduction, especially in C3 plants (Brouder and Volenec, 2008). In the present study, not only e[CO_2_], but also P supply may improve WUE by a strong interaction with CO_2_ enrichment (*P* = 0.010).

Increased WUE induced by e[CO_2_] was due to an increase in dry weight rather than a decrease in water loss (**Figures 2** and **5**). Leaf area was slightly larger in e[CO_2_] than in a[CO_2_] at 0.4, 0.8, and 2.4 P supply levels and similar among the remaining treatments (**Figure 6A**) while P levels had a major effect on this parameter. These results likely indicate that the effect of CO_2_ enrichment on leaf area was weaker than that of P supply level, although both main effects as well as their interaction were significant. However, a larger leaf area under e[CO_2_] without a concomitant increase in water loss may imply a decrease in stomatal conductance in potato plants (**Figure 6**). By contrast, P fertilization may help optimize WUE by adjusting both stomatal conductance and stomatal density. We measured productive WUE (i.e. dry matter production per unit water consumption), which is more informative for agricultural and ecological purposes, than instantaneous WUE (Sinclair et al., 1984). Here, stomatal conductance decreased under e[CO_2_] and also with increasing P supply rate (**Figure 6C**). Similarly, stomatal density was also responsive to P supply rate (**Figure 6B**), although it remained unaffected by CO_2_ enrichment. It is likely that potato plants may save water by decreasing stomatal conductance while simultaneously enhancing gas exchange by increasing stomatal density at P supply rates, thereby ultimately improving WUE. However, the relationship between stomatal density, conductance, and water use under different P supply regimes may be complex and should be examined in future investigations.

## Conclusions

The present study aimed at clarifying the following aspects: 1) to what extent can maximum biomass accumulation be enhanced by e[CO_2_] in potato plants?, 2) how much P is required to achieve maximum biomass accumulation and will e[CO_2_] increase the plant P requirement?, and finally 3) how does e[CO_2_] affect water consumption by the plant to reach maximum biomass accumulation under varying P supply? After carrying out trials we found 1) a 1.5-fold maximum biomass increase under e[CO_2_], relative to biomass accumulation under a[CO_2_]. However, 2) P requirement for maximum potato plant growth was not affected by CO_2_ enrichment as shown by a similar critical [P], and 3) total water consumption was not affected by [CO_2_] regardless of great differences in plant total biomass because of higher WUE.

The interaction between [CO_2_] and P supply provides a sound theoretical basis for P fertilizer management under e[CO_2_] conditions. As the response to CO_2_ enrichment may vary with different growing conditions and plant species (Jin et al., 2015), further research is needed to elucidate the mechanism underlying the interaction between [CO_2_] and P supply. The phosphorus crisis around the world is becoming an increasingly serious problem (Vaccari, 2009); therefore, to avoid wasteful and environmentally harmful P application in agricultural soils, and to maximize plant P-acquisition efficiency, a more sustainable paradigm in P fertilizer management should be devised, accounting for climate change, including localized-P supply (Kume et al., 2006), and arbuscular mycorrhizal fungi symbiosis (Smith et al., 2011).

## Data Availability Statement

All datasets for this study are included in the article/**Supplementary Material**.

## Author Contributions

YY and KY designed the experiment. YY conducted the experiment and collected data for analysis. YY prepared the manuscript. KY and DS revised the manuscript.

## Funding

This study was supported by JSPS KAKENHI Grant number 16H05055.

## Conflict of Interest

The authors declare that the research was conducted in the absence of any commercial or financial relationships that could be construed as a potential conflict of interest.
