# Supplementary material for: Quantifying Phosphorus and Water Demand to Attain Maximum Growth of Solanum tuberosum in a CO2-Enriched Environment
Source: Front Plant Sci. 2019 Nov 5;10:1417. doi: 10.3389/fpls.2019.01417 (PMC6848731; doi:10.3389/fpls.2019.01417)
Supplement: Supplementary file 1 [file DataSheet_1.pdf]

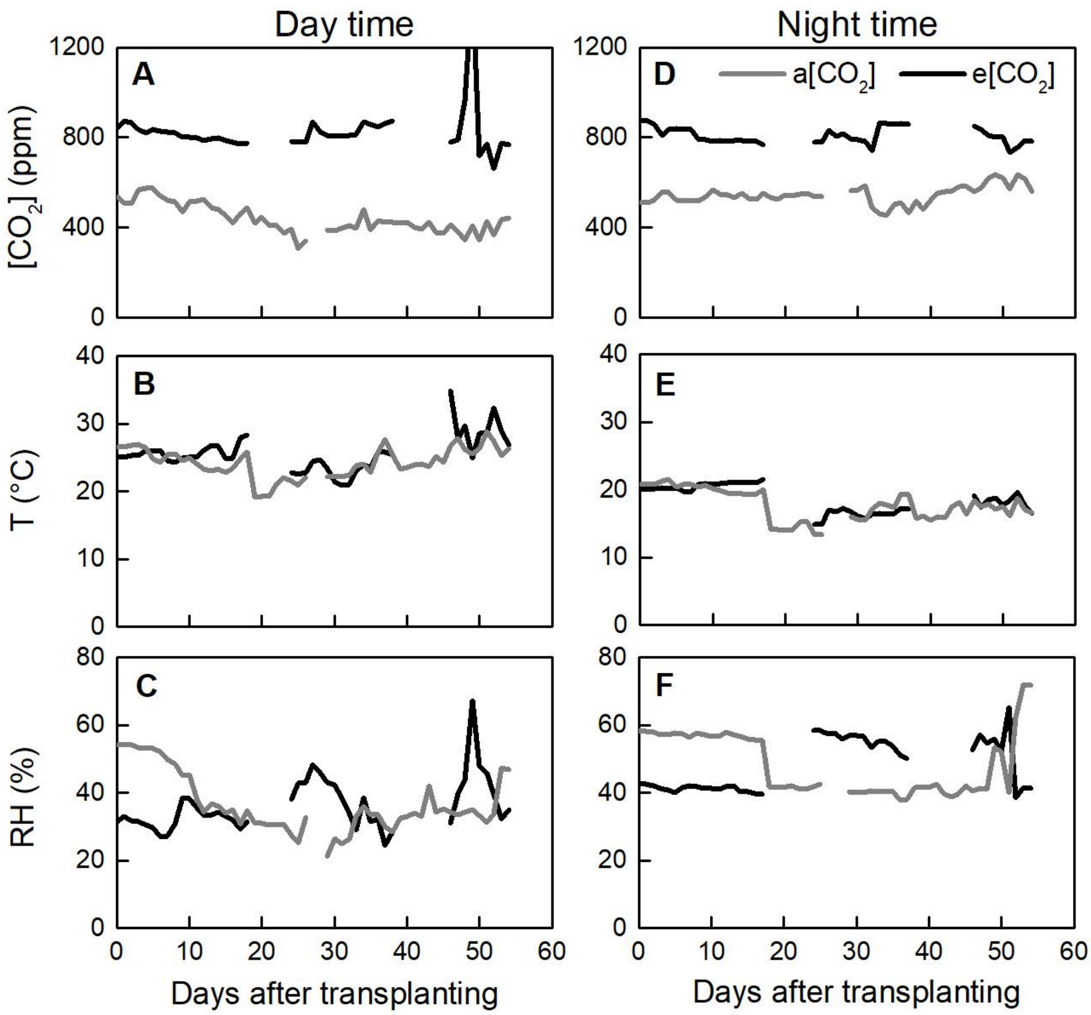

Supplementary Figure S1. Actual CO_2 concentration ([CO_2]) **(A) (D)**, temperature (T) **(B) (E)** and relative humidity (RH) **(C) (F)** in the growth chambers during the experimental period (54 days after transplanting). Missing data was unavailable due to malfunction of recorders.

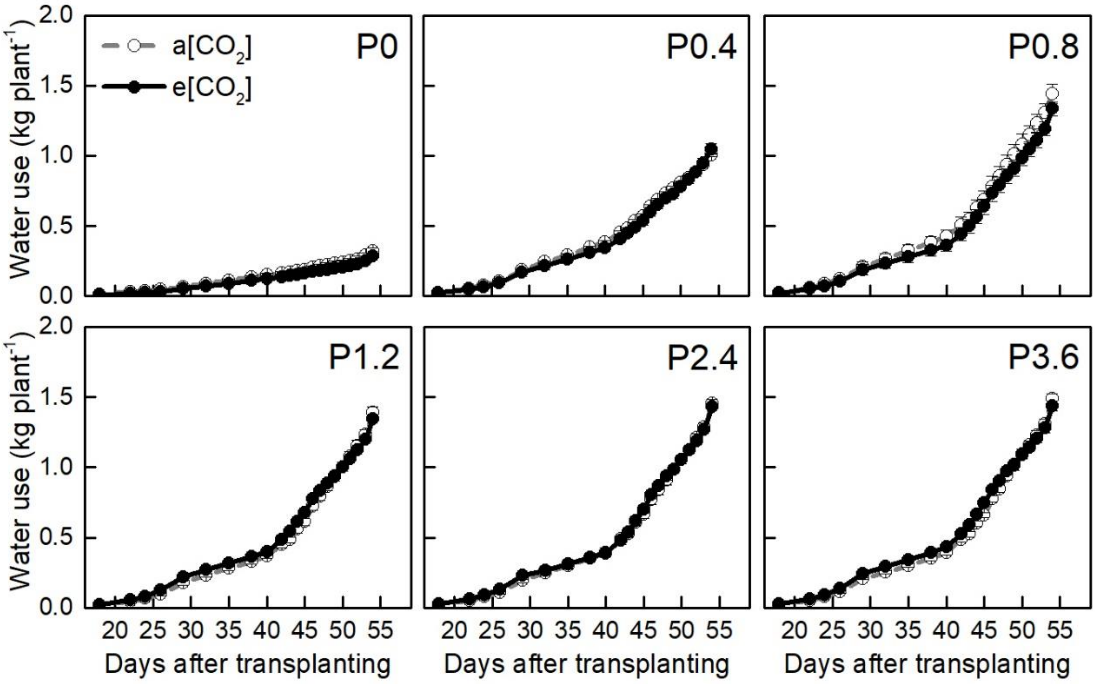

Supplementary Figure S2. Water use by potato plants over the experimental period (54 days after transplanting) under a[CO_2] (439 ± 9 ppm) and e[CO_2] (825 ± 17 ppm) at different P supply rates (0, 0.4, 0.8, 1.2, 2.4, and 3.6 g P kg^{-1} soil). Data in each plot are means ± S.E. (n = 6 or 5 biological replicates for each treatment).

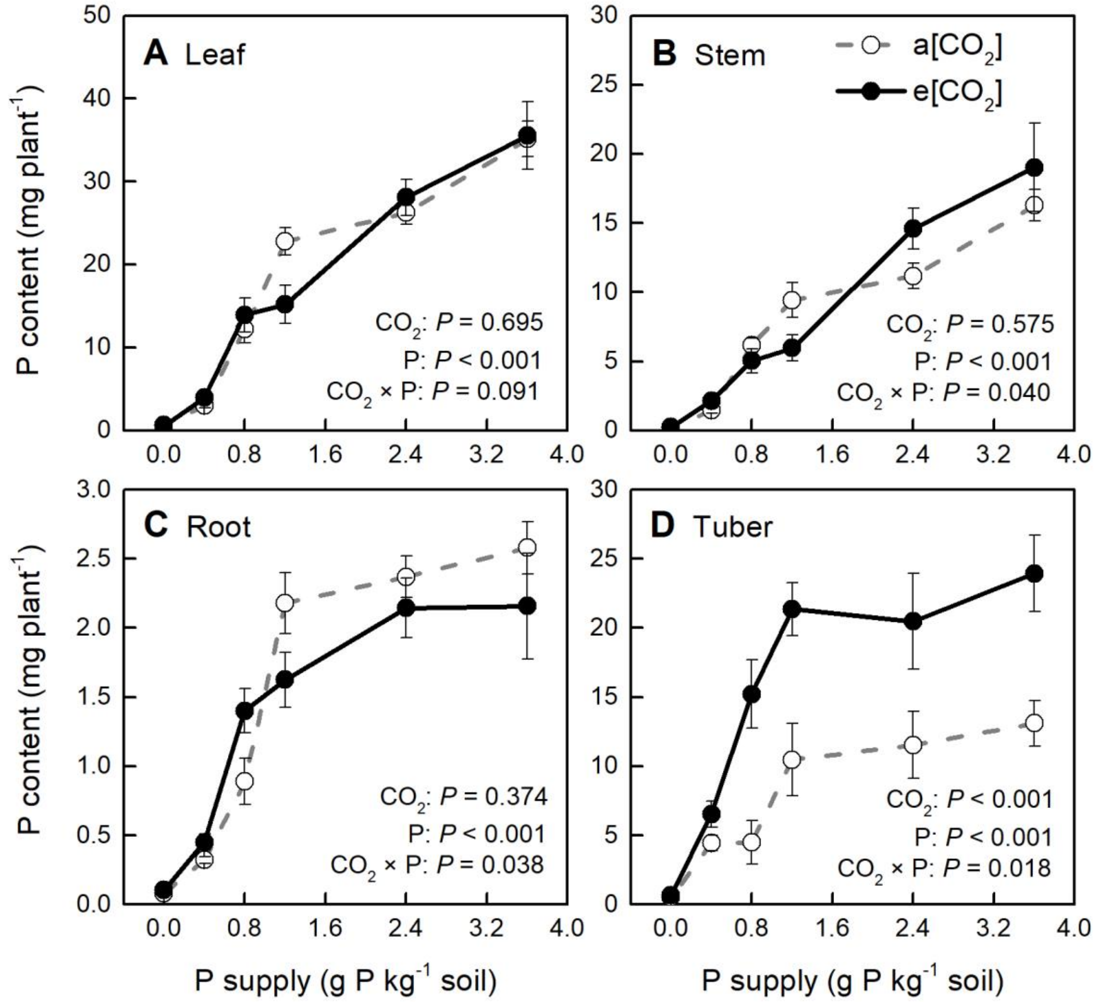

Supplementary Figure S3. P content in different organs of potato plants grown under a[CO_2] (439 ± 9 ppm) and e[CO_2] (825 ± 17 ppm) at different P supply rates (0, 0.4, 0.8, 1.2, 2.4, and 3.6 g P kg^{-1} soil). Data in each plot are means ± S.E. (n = 6 or 5 biological replicates for each treatment). Statistical comparisons (two-way ANOVA) between CO_2 concentrations and P supply rates as well as their interaction (CO_2 × P) are presented. **(A)** Leaf P content; **(B)** stem P content; **(C)** root P content; **(D)** tuber P content.

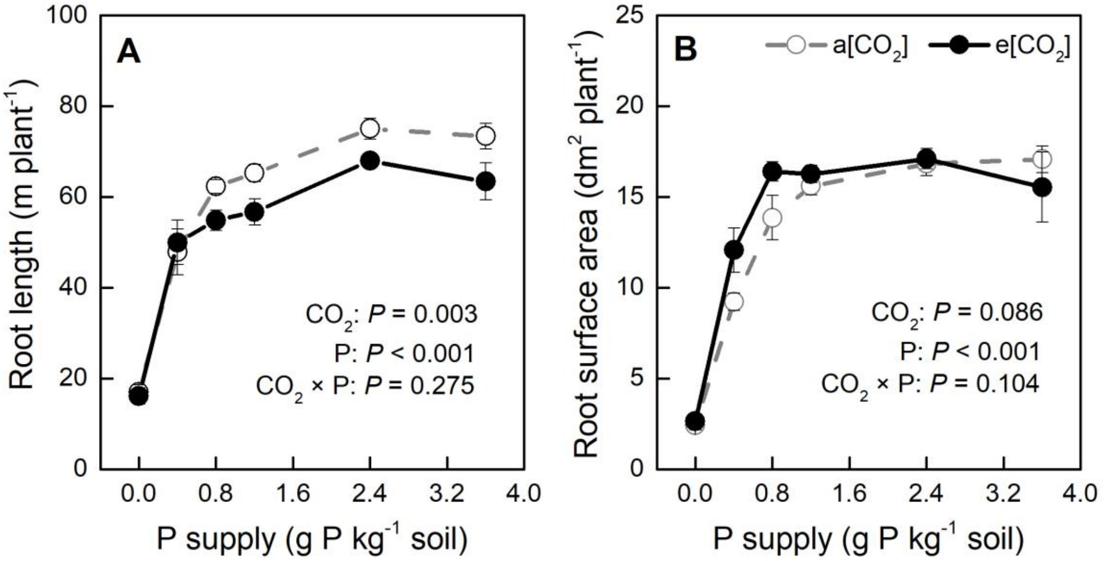

Supplementary Figure S4. (A) Root length and **(B)** root surface area in potato plants grown under a[CO_2] (439 ± 9 ppm) and e[CO_2] (825 ± 17 ppm) at different P supply rates (0, 0.4, 0.8, 1.2, 2.4, and 3.6 g P kg^{-1} soil). Data in each plot are means ± S.E. (n = 6 or 5 biological replicates for each treatment). Statistical comparisons (two-way ANOVA) between CO_2 concentrations and P supply rates as well as their interaction (CO_2 × P) are presented.
